# Surgery-related factors predisposing to uterine niche after cesarean section: a systematic review

**DOI:** 10.3389/fmed.2026.1838075

**Published:** 2026-10-12

**Authors:** Natalia Katarzyna Mazur-Ejankowska, Anna Abacjew-Chmylko, Maciej Ejankowski, Dariusz Grzegorz Wydra, Magdalena Emilia Grzybowska

**Affiliations:** Department of Gynecology and Obstetrics, Medical University of Gdańsk, Poland

**Keywords:** cesarean scar defect, cesarean scar disorder, cesarean section, hysterotomy, isthmocele, surgical technique, uterine closure, uterine niche

## Abstract

Cesarean section is among the most common surgical procedures worldwide, with rates exceeding 48% in some European countries and continuing to rise for both primary and repeat cases. Multiple cesarean deliveries have become routine in many developed countries. This trend has increased the incidence of intrapartum and long-term complications, particularly cesarean scar defects, which are symptomatic in up to 40% of affected women. A cesarean scar defect, also known as a uterine niche or isthmocele, is one of the most critical conditions in modern obstetrics and gynecology, with continuously increasing prevalence, as 56%–84% of women who undergo cesarean section develop a niche. Judicious indication for cesarean delivery and an appropriate uterine suturing technique are key factors in minimizing post-cesarean complications, including uterine niches. Uterine niche epidemiology and risk factors are widely investigated with partially contradictory conclusions. In this systematic review, a detailed analysis of surgery-related factors connected to uterine niche formation is presented. Aspects of uterine suturing technique, including the number of suture layers, locked or unlocked suturing, inclusion of the endometrium, and the choice of suture material, are compared. The aspect of single- and double-layer uterine closure remains under debate, as most studies do not show the superiority of either technique with regard to uterine niche incidence. Optimizing the hysterotomy surgical technique may decrease the rate of cesarean scar defects and their complications, including pelvic pain, abnormal uterine bleeding, dyspareunia, placenta accreta spectrum disorders, and cesarean scar pregnancies. All patients undergoing cesarean delivery should be screened for cesarean scar defects and informed about the potential complications of cesarean section.

## 1 Introduction

Cesarean section (CS) is a lifesaving procedure and one of the most commonly performed surgical procedures worldwide (1, 2). Its rate continues to increase for both primary and repeat deliveries. In 2015, an analysis of data from 169 countries covering 98.4% of global births estimated that 29.7 million births (21.1%) occurred by CS, nearly double the 12.1% recorded in 2000 (2). Regional disparities persist, ranging from 44.3% CS rate in Latin America and the Caribbean to 4.1% CS rate in West and Central Africa (2).

In 2016, the World Health Organization supported the recommendation that a CS rate of approximately 10% may be optimal, as no additional reduction in maternal or neonatal mortality was observed above this threshold (3).

A 2021 projection estimated that the global CS rate would reach 28.5% (38 million procedures) by 2030 (4). However, this projected rate had already been exceeded by 2024 in several European countries, including Poland (55.1%), Turkey (54.8%), and Greece (48.6%) (5). In 2025, England reported a CS rate of 45%, exceeding the proportion of spontaneous vaginal (44%) and instrumental vaginal births (11%) for the first time (6).

In line with the rise in CS rates, women with a history of one or multiple cesarean sections increasingly represent the predominant patient population in the delivery wards, leading to an increase in intrapartum and long-term complications, with cesarean scar defect (CSD) at the forefront (7). CSD also known as a uterine niche or isthmocele, is defined as an indentation of the myometrium at the site of a uterine hysterotomy incision with a depth of at least 2 mm, and it is one of the most timely cesarean section long-term complications (8). CSD is detected in 56%–84% of women post CS and may result in life-threatening conditions of cesarean scar pregnancy and placenta accreta spectrum (PAS) disorders in subsequent pregnancies (9). In non-pregnant women, the uterine niche may remain asymptomatic; however, up to 40% of women with CSD experience spotting and abnormal uterine bleeding (AUB), dyspareunia, dysmenorrhea, chronic pelvic pain, and secondary infertility, substantially impairing their quality of life (10).

Although CSD epidemiology and risk factors have been extensively investigated, the role of surgical technique remains incompletely understood. Proposed determinants include surgical factors as well as patient- and labor-related characteristics, including body mass index (BMI), medical history, stage of labor, and cervical dilation at the time of CS.

The aim of this systematic review is to summarize the available evidence on the association between cesarean surgical technique and CSD formation, focusing specifically on single- vs. double-layer uterine closure, locked vs. unlocked suturing, endometrial inclusion vs. exclusion, knot-tying and tissue-apposition methods, and the selection of suture materials.

### 1.1 Niche evaluation

A consensus on ultrasonographic niche evaluation was reached by an expert panel using the modified Delphi method, wherein a uterine niche was defined as an indentation at the site of a cesarean scar of at least 2 mm in depth (8). Complete measurements of the niche include: length, depth, residual myometrial thickness (RMT), width, adjacent myometrial thickness (AMT), distance between the niche and the vesicovaginal fold, and distance between the niche and the external cervical os. The niche length and depth, RMT, and AMT should be measured in the sagittal plane (Figure 1), whereas niche width is measured in the transverse plane (8). Transvaginal ultrasonography (TVUS), gel infusion sonohysterography (GIS), saline infusion sonography (SIS), and magnetic resonance imaging (MRI) can all be used for niche imaging to enable diagnosis (11).

**Figure 1:**
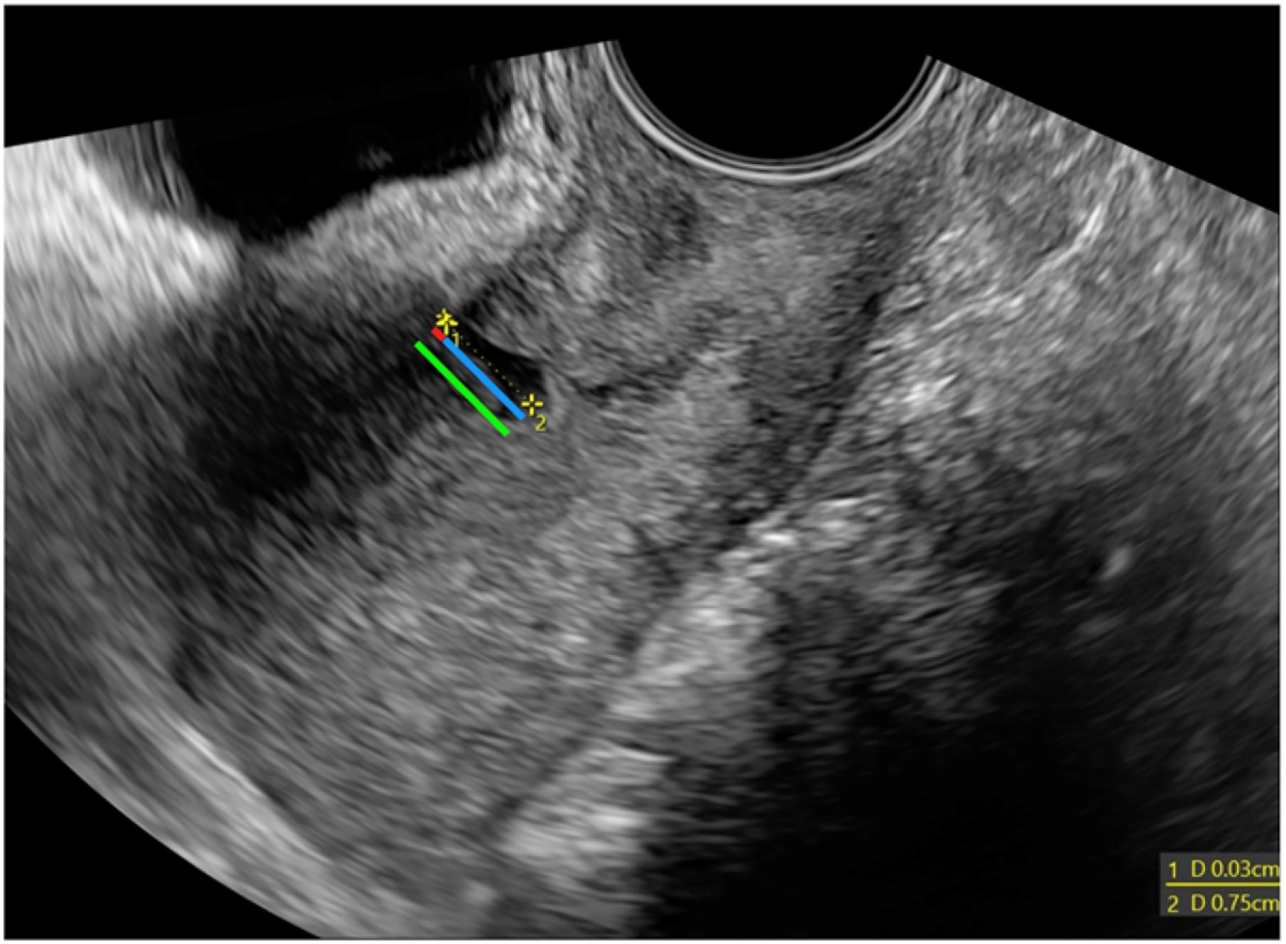
Transvaginal ultrasonography image of uterus with cesarean scar defect. Red line marks RMT, length of 0.03 cm (distance 1). Blue line marks niche depth, length of 0.75 cm (distance 2). Green line marks AMT.

## 2 Materials and methods

The systematic review was conducted and reported in accordance with the PRISMA 2020 guidelines (12) and cross-checked by two authors (NM-E and ME) from inception to January 2026 on different platforms, including PubMed, Scopus, and clinicaltrials.gov. The search string included terms “cesarean niche,” “uterine niche,” “cesarean scar defect,” and “isthmocele” and revealed 426 entries. Six additional relevant articles analyzing the risk factors for developing a uterine niche were sourced from cross-referencing. After the removal of duplicates, case series/reports and reviews, original articles were further analyzed. Finally, relevant, high-quality (peer-reviewed), original articles available in full text in English were included for an in-depth analysis. Studies not related to CS surgical technique and its influence on CSD formation were excluded from this review. Studies related to CS operative technique and CS suturing material were included in the review (Figure 2).

**Figure 2:**
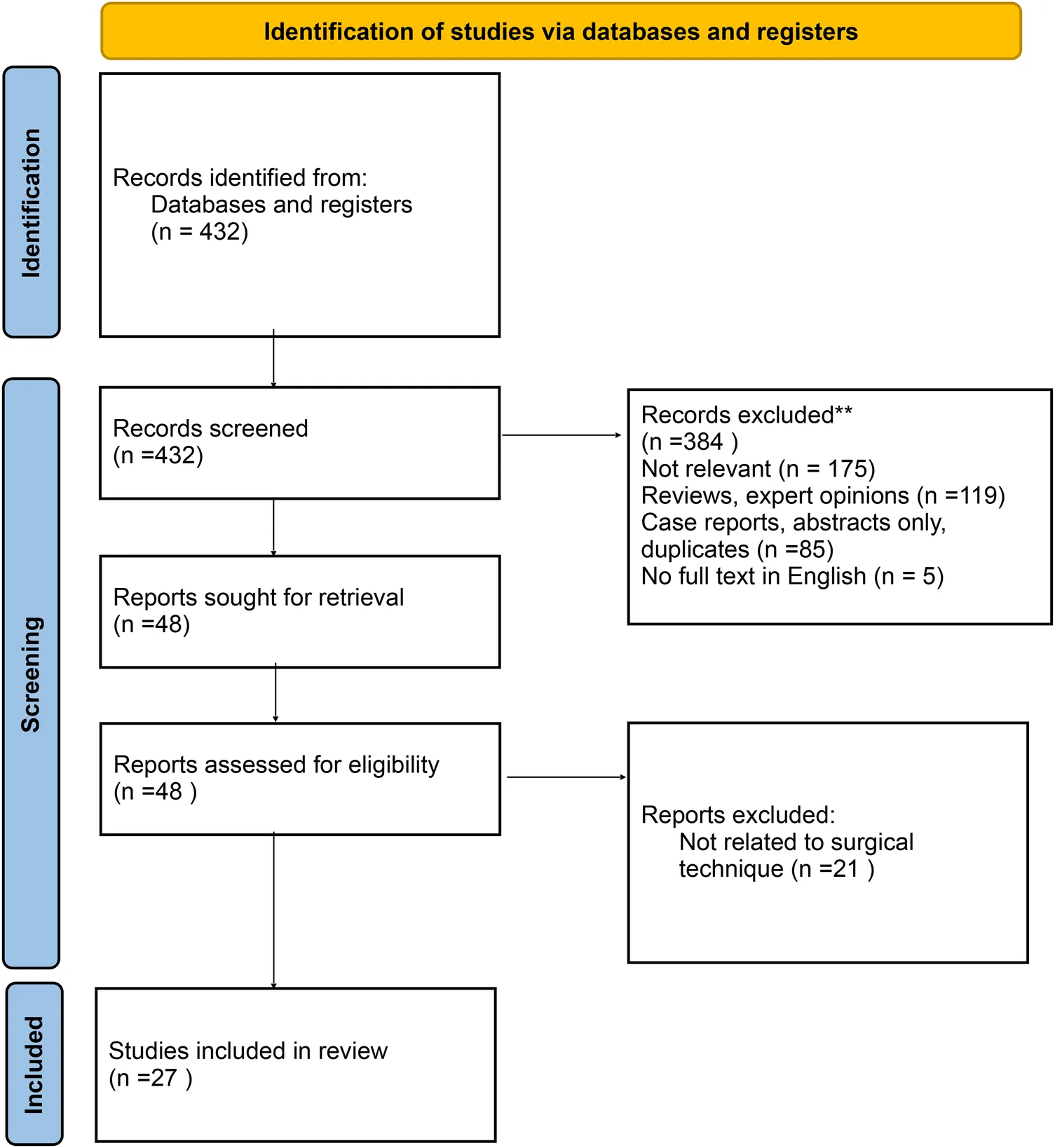
PRISMA 2020 flow diagram for systematic reviews included searches of databases and registers only.

The formation of the uterine niche is considered a multifactorial process, and efforts were made to divide the risk factors into separate categories for maintaining clarity of the review. Articles investigating CS surgical techniques were divided into five subgroups: (1) single-layer and double-layer uterine closure techniques (12 papers); (2) suturing material (6 papers); (3) Endometrium-free and non-endometrium-free uterine closure (4 papers); (4) locked and unlocked suturing technique (4 papers); (5) other uterine closure techniques (3 papers). Some articles were included in more than one subgroup due to multiple risk factors analyzed. Studies using a uterine niche definition consistent with the modified Delphi method consensus were included in this review.

Twenty studies, including randomized clinical trials (RCTs) and randomized prospective studies, were evaluated using the Cochrane Risk of Bias 2.0 (RoB 2.0) tool. Three studies (21, 29, 36) were assessed as having a high risk of bias, mainly due to high attrition rates and limited information about the allocation concealment process. The rest of the studies were assessed as some concern of bias or low risk of bias (Appendix A, Table A1). The Newcastle–Ottawa Scale (NOS) was used to assess the risk of bias and quality of retrospective and cohort studies discussed in the review (Appendix A, Table A2). All studies were assessed as high quality, scoring at least seven out of nine points on the NOS. The limitations of some studies included non-randomized intervention allocation, residual confounding, and possible selection bias. In addition, the risk of bias may be increased due to the single-center character of the studies. This systematic review was not prospectively registered, and no publicly accessible review protocol is available. This should be considered a methodological limitation when interpreting the findings.

## 3 Results

### 3.1 Single-layer and double-layer uterine closure

The CORONIS (13) trial is the largest to date RCT conducted across multiple continents on 15,935 patients after cesarean section. The study compared different aspects of the cesarean section technique, including single-layer and double-layer uterine closure, with regard to maternal mortality and infectious morbidity, further operative procedures, and blood transfusion rates until the 6-week postpartum home visit or telephone interview. Both single-layer and double-layer uterine closures were deemed acceptable as no statistically significant differences in the study outcomes [risk ratio (RR) 0.96; 95% confidence interval (CI) 0.85–1.08; *p* > 0.05]; however, no imaging studies were performed in this study. In the CORONIS follow-up study (14), conducted after 3.8 years, no evidence of statistically significant differences were observed in maternal death (RR 0.78, CI 0.46–1.32) or a composite of pregnancy complications (RR 1.20, CI 0.75–1.90) between single-layer and double-layer closure of the uterus. The authors underlined that the study was not powered to detect modest differences but serious events, which is a limitation. The CORONIS trial concluded that there was no evidence to favor one cesarean section technique over another, which influenced cesarean delivery recommendations by the World Health Organization (WHO) (15).

The 2Close RCT (16) was carried out in 2,292 women undergoing primary CS to compare single-layer and double-layer uterine closure techniques. This study included a follow-up scheduled at 3 months post-CS when TVUS, GIS, or SIS was performed to assess the uterine scar. At three months postoperatively, the authors reported a uterine niche prevalence of 68.9% after single-layer uterine closure, compared with 73.6% after the double-layer technique, which was a statistically significant difference (adjusted RR 1.06, 95% CI 1.01–1.13, *p* = 0.033). The operative time was longer in the double-layer group; adjusted mean difference of 3.9 min (95% CI 3.0–4.9, *p* < 0.001). The proportion of women needing treatment for gynecological complaints was significantly higher after double-layer closure (2.8%) than after single-layer closure (1.4%) when considering the risk difference of 0.02 (95% CI 0.00–0.03, *p* = 0.04), but this difference did not reach significance when considering the relative risk of 1.93 (95% CI 0.99–3.75, *p* = 0.052). No significant differences were found in the prevalence of large niches defined as RMT of less than 3 mm or RMT less than 50% of the AMT or in other sonographic secondary outcomes (16).

After the initial 2-year study, a 3-year follow-up questionnaire study was conducted with 73.7% of patients responding in the single-layer and 72.9% in double-layer closure. The results did not demonstrate the superiority of double-layer closure over single-layer closure with regard to reproductive outcomes such as live births and pregnancy rates, fertility treatment rates, mode of delivery, and uterine ruptures in subsequent pregnancies (17).

The most recent NICEST study (18), conducted in 2025, was a RCT of 530 patients after primary CS, comparing RMT and niche incidence and characteristics between single- and double-layer uterine closure, assessed at two TVUS follow-up visits at 6 and 12 months post-CS. The niche incidence was similar between the single-layer (35.6%) and double-layer (31.9%) (*p* > 0.05). However, RMT was significantly greater in the double-layer than in the single-layer group (4.3 vs. 4.0 mm; *p* = 0.007), and a lower proportion of large niches with residual myometrial thickness of <3 mm was detected in the double-layer group compared with the single-layer group (9.9% vs. 19.4%; *p* = 0.033). The authors additionally concluded that uterine scar characteristics were stable at 6 months after cesarean delivery, but the niche volume significantly increased over time as assessed at 12 months post-CS.

A retrospective cohort study on 149 women investigated uterine niche rates after single-layer and double-layer hysterotomy closure following first elective CS. At a 6-month follow-up TVUS, the median RMT was 5.8 mm in women with double-layer closure vs. 4.6 mm in single-layer closure, a statistically significant difference (*p* = 0.04). Scar defect length was significantly greater in single-layer closure than in double-layer closure (*p* = 0.01). The authors concluded that their results indicate a limited benefit of double-layer closure following first elective CS (19). Nine years later, Glavind et al. (20) published the results of their second study, an RCT of 230 women with a follow-up at 6 months post-CS. The results were in contrast to the first study, as a uterine niche was observed in 52% of women after the double-layer technique and 43% of women after single-layer closure (95% CI 6.9–24.2), and the niches were deeper after the double-layer technique than after single-layer closure (95% CI 0.01–1.02). The authors concluded that the single-layer technique was associated with an absolute 8.6% decrease in the risk of having a uterine niche with a depth >2 mm 6 months after delivery compared to a double-layer closure; however, the difference was not statistically significant.

An RCT by Bamberg et al. of 435 women found no significant differences in niche incidence or depth between single- and double-layer closure at 2-year follow-up. However, residual myometrial thickness tended to be greater after double-layer closure (*p* = 0.06) (21).

In three Turkish prospective studies involving women who underwent primary CS, the authors reported that the incidence of niche and its depth were independent of the hysterotomy closure technique, with no statistically significant difference between single-layer and double-layer uterine closure regarding the length and depth of the niche after 6 weeks to 9 months follow-up (22–24).

Two studies favored the double-layer hysterotomy closure techniques (25, 26). A 2022 RCT of 100 women by Dimassi et al. (25) found a significant decrease in the rate of CSD in the double-layer purse-string uterine suture (PSUS) group compared to the single-layer continuous uterine suture (SLCUS) group (6.66% vs. 40%) on SIS at 6 months. In a study published in 2024, the niche rates were 20.6% vs. 60.7%, respectively (26). Despite a longer operative time with double-layer closure, there was no significant difference in blood loss (25).

On the other hand, a 2023 RCT by Halouani et al. (27) revealed that despite no difference in residual myometrial thickness, purse-string uterine closure seems to be associated with better uterine scar healing and a lower rate of cesarean scar defects than double-layer continuous unlocked suture.

The CAESAR group analyzed single- and double-layer uterine closure, closure and non-closure of the pelvic peritoneum, and liberal vs. restricted use of a subrectus sheath drain on 3,033 women after first CS (28). There were no differences between the arms of the trial for the primary results, that is, maternal infectious morbidity reported until 6 weeks postpartum. There were no differences in any of the secondary morbidity outcomes and no signiﬁcant adverse effects of any of the techniques used. The authors concluded that the lack of differences between the interventions in the short term may suggest that any one of the three pairs of approaches is acceptable (28) (Table 1).

**Table 1:** Comparison of studies describing single-layer and double-layer uterine closure.

Author/name of study	Year	Study type	Qualification criteria	Number of patients	Time of assessment after CS	Method of assessment	Study objective	Results	Conclusions
Nicest study group (18)	2025	RCT	One CS	530	6 and 12 months	TVUS	Comparing single-layer and double-layer uterine closure	Uterine niche incidence: single layer, 35.6% double layer, 31.9% 6 months after CS: Double layer resulted in greater RMT (4.3 vs. 4.0 mm; *p* = 0.007) Double layer resulted in a lower proportion of large niches with RMT of <3 mm (9.9% vs. 19.4%; *p* = 0.033).	Similar niche incidence in single- and double-layer closure Superiority of double layer: greater RMT, lower large niche proportion, and smaller niche volume
2Close study group (16, 17)	2024	RCT	One CS	2,292	3 months	TVUS or GIS/SIS	Comparing single-layer and double-layer uterine closure	Uterine niche incidence: single layer, 68.9% double layer, 73.6% No significant differences were found in: RMT and the prevalence of large niches	Statistically more niches after double-layer closure (RR 1.06, 95% CL 1.01–1.12, *p* = 0.033) The operative time was longer in the double-layer group Women needing treatment for gynecological complaints: single layer, 1.4% double layer, 2.8% (significantly higher)
Mehdiyev et al. (26)	2024	Randomized prospective	One or more CS	57	6 months	TVUS	Comparing single-layer and double-layer uterine closure	Uterine niche incidence: single layer, 60.7% double layer, 20.6%	Double-layer closure can be used to reduce the risk of uterine niche development
Yıldız and Timur (24)	2023	Prospective	One CS	111	6 weeks	TVUS	Comparing single-layer and double-layer purse-string uterine closure	Uterine niche incidence: single layer, 20.8% double purse-string layer, 10.3%	Niche incidence was not significantly different between single layer and double purse-string technique (*p* = 0.128)
Halouani et al. (27)	2023	RCT	One CS	126	6 months	SIS	Comparing purse-string and double-layer uterine closure	Uterine niche incidence: single purse-string layer, 12% double purse-string layer, 35% No significant difference between the groups in terms of residual myometrial thickness, blood loss, or operative time	Purse-string uterine sutures allowed significantly fewer cesarean scar defects (*p* = 0.018) Achievement of purse-string uterine suture required significantly fewer threads and fewer hemostatic complementary sutures
Dimassi et al. (25)	2022	RCT	One or more CS	100	6 months	SIS	Comparing single-SLCUS and double-layer PSUS	Uterine niche incidence: single layer (SLCUS), 40% double layer (PSUS), 6.66% Despite a longer operative time with PSUS, there was no significant difference in terms of blood loss	Compared to SLCUS, the PSUS significantly reduces uterine niche rates, without increasing the perioperative maternal morbidity
Yılmaz Baran et al. (23)	2018	Prospective, randomized	One CS	282	6–9 months	SIS	Comparing single-layer and double-layer uterine closure	Uterine niche incidence: single purse-string layer, 37% double purse-string layer, 45.7%	Single- and double-layer closure techniques were not statistically significant on uterine niche rates
Tekiner et al. (22)	2018	Prospective cross-sectional	One CS	280	3 months	SIS	Comparing single-layer and double-layer uterine closure	No statistically significant difference between single- and double-layer groups regarding RMT, length, and depth of uterine niche (*p* = 0.70)	Single- or double-layered uterine closure does not seem to affect niche size
Bamberg et al. (21)	2017	RCT	One or more CS	435	6 weeks, 6–24 months	TVUS	Comparing single-layer unlocked, single-layer locked, and double-layer uterine closure	Uterine niche incidence in 1st follow-up: single-layer unlocked, 40% single-layer locked, 32% double-layer sutures, 43% Uterine niche incidence in 2nd follow-up: single-layer unlocked, 30% single-layer locked, 23% double-layer sutures, 29%	Uterine niche incidence and depth were not significantly different after single- or double-layer closure in 2 years follow-up There was a trend (*p* = 0.06, not statistically significant) for RMT to be greater after double-layer repair
Glavind et al. (19)	2013	Retrospective cohort	One CS	149	6 months	TVUS	Comparing single-layer and double-layer uterine closure	Uterine niche incidence: single layer, 94% double layer, 64% Median RMT was 5.8 mm in women with double-layer closure vs. 4.6 mm in single-layer closure	Uterine niche was significantly higher in the single-layer compared to double-layer closure (*p* = 0.01) RMT was statistically greater after double-layer closure (*p* = 0.0.4)
Glavind et al. (20)	2023	RCT	One CS	230	6 months	SIS	Comparing modified single-layer alternating deep-superficial stitches and double-layer uterine closure	Uterine niche incidence: single layer, 43% double layer, 52% The niche was deeper with double-suture (mean 2.5 mm) vs. single-suture (mean 2.0 mm) with a risk difference of 0.52 mm (95% CI 0.01–1.02)	Single-layer closure was associated with an absolute 8.6% decrease in the risk of uterine niche with a depth >2 mm 6 months after delivery compared to a double-suture closure (not statistically significant)
CORONIS group (13, 14)	2013	RCT	One or two CS	15,935	Immediately after CS, 6 weeks	Telephone interview or home visit	Comparing single-layer and double-layer uterine closure and blunt vs. sharp abdominal entry; exteriorization of uterus for repair vs. intra-abdominal repair; closure vs. non-closure of the peritoneum (pelvic and parietal); and chromic catgut vs. polyglactin-910 for uterine repair	The primary outcome was the composite of death, maternal infectious morbidity, further operative procedures, or blood transfusion (>1 unit) up to the 6 week follow-up visit, which revealed that all compared techniques are acceptable	There were no statistically significant differences within any of the intervention pairs for the primary outcome: blunt vs. sharp entry, risk ratio 1.03 (95% CI 0.91–1.17); exterior vs. intra-abdominal repair, 0.96 (0.84–1.08); single-layer vs. double-layer closure, 0.96 (0.85–1.08); closure vs. non-closure, 1.06 (0.94–1.20); and chromic catgut vs. polyglactin-910, 0.90 (0.78–1.04)
CAESAR group (28)	2010	RCT	One CS	3,033	6 weeks	Postal questionnaire	Comparing single-layer and double-layer uterine closure, closure and non-closure of the pelvic peritoneum, and liberal and restricted use of a subrectus sheath drain	The main outcome measure was maternal infectious morbidity up to the 6-week follow-up visit: no statistical differences between the arms of the trial were noted There were no differences in morbidity outcomes and no signiﬁcant adverse effects of any of the techniques used	The lack of differences between the interventions in the short term may suggest that any one of the three pairs of approaches is acceptable.

CS, cesarean section; GIS, gel infused sonography; PSUS, purse-string uterine suture; RCT, randomized control trial; RMT, residual myometrial thickness; SIS, saline infused sonography; SLCUS, single-layer continuous uterine suture; TVUS, transvaginal ultrasound.

### 3.2 Using locked and unlocked suturing technique

The locked and unlocked suturing techniques for the post CS hysterotomy closure were investigated by four authors who conducted two RCTs and two prospective, randomized studies. It is hypothesized that the locked technique may impair the wound vascularization by causing restrictive blood flow to the uterine scar (Table 2).

**Table 2:** Comparison of studies describing using locked vs. unlocked suturing techniques.

Author/name of study	Year	Study type	Qualification criteria	Number of patients	Time of assessment	Method of assessment	Study objective	Results	Conclusions
Gumusburun and Uskent (31)	2025	Prospective, randomized	One CS	120	3 months post CS	TVUS	Comparing uterine closure techniques: single-locked, single-unlocked and baseball	Uterine niche incidence: single-locked layer, 47.5% single-unlocked layer, 46.2% baseball closure, 15.4%	Compared with single-locked and single-unlocked groups, uterine niche development was statistically significantly lower in the baseball-type closure group (*p* = 0.004)
Bayraktar et al. (30)	2022	Randomized, prospective	One or more CS	194	6 months post CS	TVUS	Comparing uterine closure techniques: single-locked and single-unlocked During the surgery, the thicknesses of LUS and UUS were measured	Uterine niche incidence: single-locked layer, 34.3% single-unlocked layer, 25.3% The mean UUS/LUS ratio is 1.4 ± 0.16 among patients without uterine niche and 2.21 ± 0.31 in patients with uterine niche (*p* < 0.001)	No statistically significant difference in niche size between single-layer locked and unlocked suturing (*p* = 0.167) The ratio of upper to lower uterine segment that was not previously investigated in literature is a major risk factor for the development of uterine niche
Tekelioglu et al. (29)	2021	RCT	Two CS	120	6–8 months post CS	TVUS	Comparing uterine closure techniques: single-locked and single-unlocked in recurrent cesarean section During the surgery, the thicknesses of the LUS and UUS were measured	Uterine niche incidence: single-locked layer, 60% single-unlocked layer, 57.5% In women with uterine niche, LUS was 4 mm thinner than in women without niche (*p* = 0.006)	There was no association between uterine niche and locked and unlocked techniques Thin LUS and unevenness between LUS and UUS may play a role in incomplete healing of the uterine incision
Bamberg et al. (21)	2017	RCT	One or more CS	435	6 weeks, 6–24 months post CS	TVUS	Comparing uterine closure technique: single-locked, single-unlocked and double layer	Uterine niche incidence (1st follow-up): single-locked layer, 32% single-unlocked layer, 40% double layer, 43% Uterine niche incidence (2nd follow-up): single-locked layer, 23% single-unlocked layer, 30% double layer, 29%	The incidence of niche formation and depth was independent of the hysterotomy closure technique Niche incidence and depth were not significantly different after single- vs. double-layer closure in 2 years follow-up There was a trend (*p* = 0.06) for the residual myometrium thickness to be thicker after double-layer repair

CS, cesarean section; CSD, cesarean scar defect; LUS, lower uterine segment; RCT, randomized control trial; RMT, residual myometrial thickness; TVUS, transvaginal ultrasound; UUS, upper uterine segment.

An RCT by Bamberg et al. (21), conducted in 2017 on a group of 435 patients who underwent two TVUS follow-up visits 6 weeks and 6–24 months postoperatively, reported that the incidence of niche formation and its depth were independent of the hysterotomy closure technique. The niche rates at the second follow-up visit were 30% for single-layer unlocked and 23% for single-layer-locked technique and were not statistically significant. Similarly, an RCT conducted by Tekelioglu et al. (29), at 6 months post-CS by TVUS, on a group of 120 patients after second CS reported that there was no association between uterine niche and locked vs. unlocked suture technique used for the closure of the uterine incision. The biometric evaluation of the scar area has shown that the thin lower wound edge and unevenness between the lower and the upper wound edges may play a role in incomplete healing of the uterine incision (29).

A randomized, prospective study by Bayraktar et al. (*n* = 194) revealed a rate of 34.3% of niches after single-layer-locked continuous suturing and a 25.3% rate after single-layer unlocked continuous suturing (*p* = 0.167) (30). During surgery, the thickness of the lower uterine segment (LUS) and upper uterine segment (UUS) was measured using a sterile scale prior to hysterotomy closure. The authors concluded that the ratio of UUS to LUS is a major risk factor for the development of a uterine niche. This is a new concept not previously investigated in the literature, which is in line with the previously discussed Tekelioglu et al. RCT results (29).

A recent prospective, randomized study (*n* = 118) by Gumusburun and Uskent (31) compared three suturing techniques. Niche incidence was 47.5% for the single-layer locked, 46.2% for the single-layer unlocked, and 15.4% for baseball-type uterine closure techniques. Hysterotomy closure with baseball-type suturing had a statistically significantly lower incidence of niches (*p* = 0.004) (31). No statistical difference was noted between the locked and unlocked single-layer uterine closures.

The authors of the four mentioned above studies did not show a statistically significant effect of unlocked vs. locked uterine closure technique with regard to uterine niche formation. However, the results of a meta-analysis of 20 studies by Stegwee et al. revealed that the single-layer-locked suturing is associated with lower RMT and an increase in isthmocele incidence compared with the unlocked technique (32).

### 3.3 Endometrium-free and non-endometrium-free uterine closure

It is hypothesized that including the endometrial layer when suturing a post-CS hysterotomy causes a disruption of the uterine architecture, and three RCTs and two retrospective studies investigated endometrium-free and non-endometrium-free uterine closure (Table 3). The disruption of uterine tissues at the cellular level, through incorporating the endometrial layer into the myometrial layer, could be one of the causes of cesarean scar defect development (33).

**Table 3:** Comparison of studies describing endometrium free vs. non-endometrium-free uterine closure.

Author/name of study	Year	Study type	Qualification criteria	Number of patients	Time of assessment	Method of assessment	Study objective	Results	Conclusions
2Close study group (16, 17)	2024	RCT	One CS	2,292	3 months post CS	TVUS or GIS/SIS	Comparing uterine closure techniques: endometrial closure vs. endometrial non-closure in single-layer uterine closure group	Uterine niche incidence: endometrial closure single layer, 71.7% endometrial non-closure single layer, 59.3%	Niche prevalence was lower after single-layer uterine closure excluding the endometrium comparing with single-layer uterine closure including the endometrium No significant differences were found in the prevalence of large niches, in RMT or in the other sonographic secondary outcomes
Mohr-Sasson et al. (37)	2024	RCT	One CS	130	6 months post CS	TVUS	Comparing uterine closure techniques with regards to primary outcome of RMT: endometrial closure vs. endometrial non-closure.	Median RMT: Endometrial closure group 5.3 mm (3.0–7.7) vs. endometrial non-closure group 4.6 mm (3.0–6.5)	Closure vs. non-closure of the endometrial layer has no significant difference (*p* = 0.38) in cesarean scar characteristics and symptom rates at 6 months follow-up
Gezer et al. (36)	2024	RCT	One CS	274	6 moths post CS	TVUS	Comparing uterine closure techniques: endometrial closure vs. endometrial non-closure	Uterine niche incidence: endometrial closure, 29.6% endometrial non-closure, 15.4% Menstrual irregularities, such as intermenstrual spotting, were significantly higher in the endometrial suturing group than in the non-endometrial	Uterine closure with non-endometrial suturing was associated with significantly lower uterine niche and less intermenstrual spotting compared to that with endometrial suturing
Meyer et al. (35)	2023	Retrospective	One or more CS	172	Unspecified	SIS	Comparing double-layer uterine closure techniques: endometrial closure (technique B) vs. endometrial non-closure (technique A)	Double-layer routine endometrial closure was associated with more clinically significant niches across all subgroups, and these niches were significantly longer and deeper when present	Endometrium-free double-layer hysterotomy closure technique may help to reduce niche development and severity
Antoine et al. (34)	2021	Retrospective	One CS	45	>6 months post CS	SIS	Comparing uterine closure techniques: endometrial closure vs. endometrial non-closure	A clinically significant niche development was six times higher with routine non-endometrium-free when compared to endometrium-free	Exclusion of the endometrium at uterine closure reduces the development of significant scar defects

CS, cesarean section; GIS, gel infused sonography; RCT, randomized control trial; RMT, residual myometrial thickness; SIS, saline infused sonography; TVUS, transvaginal ultrasound.

In a retrospective study of 45 women with a history of CS, clinically significant niche development was six times higher with routine non-endometrium-free closure when compared with endometrium-free closure, assessed by SIS (34).

It was confirmed by Meyer et al. that endometrium-free closure is superior and should be used routinely during first and subsequent cesarean sections even when the lower uterine segment is thin (35).

The previously mentioned large RCT 2Close on 2,292 post-CS patients compared the endometrial handling during hysterotomy. At the 3-month gynecological follow-up TVUS, GIS or SIS was performed to uncover rates of uterine niches. The endometrial handling technique was recorded for 1,036 women: 71.7% of women received uterine closure including the endometrium, and 28.3% of women received uterine closure excluding the endometrium. The prevalence of uterine niche formation was lower after single-layer uterine closure excluding the endometrium (59.3%) compared with single-layer uterine closure including the endometrium (71.8%) (adjusted RR 0.83, 95% CI 0.74–0.93, *p* = 0.001) (16, 17).

The results of two other RCTs published in 2024 included patients undergoing their first cesarean section who were examined by TVUS at 6-month post-CS follow-up (35, 36). Gezer et al. (36) concluded that endometrium-free uterine closure was associated with significantly lower uterine niche development (15.4% vs. 29.6% for non-endometrium-free closure) and significantly less intermenstrual spotting compared to that with endometrial suturing (*p* = 0.019). On the other hand, Mohr-Sasson et al. (37) concluded that closure vs. non-closure of the endometrial layer during cesarean uterine incision repair has no statistically significant difference in cesarean scar characteristics and symptom rates at the 6-month follow-up.

### 3.4 Other suturing techniques

Three papers present novel techniques developed for post-CS hysterotomy closure (38–40) as single-center, original studies, which signals a potential preliminary risk of bias. These studies compared traditional suturing with newly described techniques, which the authors tested with regard to uterine niche rates, post-CS symptoms, and operative time (Table 4). Kalem et al. (38) compared two uterine closure techniques: a double-layer, far-far-near-near (FFNN) unlocked technique and a single-layer continuous locked (SLL) technique in 138 patients during the first CS. A TVUS performed at a follow-up 6 months post-CS revealed that uterine niche formation was less frequent and RMT was greater in the FFNN group (*p* < 0.001).

**Table 4:** Comparison of studies describing other uterine closure techniques.

Author/name of study	Year	Study type	Qualification criteria	Number of patients	Time of assessment	Method of assessment	Study objective	Results	Conclusions
Tsuji et al. (40)	2025	RCT	One or more CS	220	6–8 months	SIS	Comparing uterine closure techniques: double-layer interrupted and double-layer continuous	Severe cesarean scar defect rates: Double-layer interrupted, 2% Double-layer continuous, 22%	Double-layer interrupted sutures are superior to continuous sutures in preventing uterine niches Other factors contributing to developing severe niches: difference in myometrial thickness between the fundal and cervical sides at the center of the uterine wound, retroversion of the uterus
Erkayıran and Arslanca (39)	2022	Prospective	One CS	402	3 months post CS	TVUS	Comparing uterine closure techniques: CPCS and NTUS comprising of Z suture on both corners and eight sutures in the remaining middle part incision closure	Only one patient with uterine niche was found in each of the groups comparing uterine closure RMT showed a negative strong correlation with incision length after closure, how many times the needles have been passed and time for suturing	There was no statistical difference between uterine niche incidence in both groups The novel suturing technique resulted in reduced operation time, decreased number of sutures, decreased blood loss and increased RMT
Kalem et al. (38)	2021	RCT	One CS	138	6 months post CS	TVUS	Comparing uterine closure techniques: double-layer, FFNN unlocked technique vs. SLL	Uterine niche rates: FFNN, 10% single layer, 41.2% Postmenstrual spotting was less common in FFNN group, while the rates of chronic pelvic pain and dysmenorrhea were not significantly different between the groups	Uterine closure using FFNN technique is beneficial in terms of providing protection from isthmocele formation and ensuring sufficient RMT

CPCS, classical primary continuous suturing; CS, cesarean section; CSD, cesarean scar defect; FFNN, double-layer far-far-near-near suturing; NTUS, novel technique uterine suturing; RCT, randomized control trial; RMT, residual myometrial thickness; SIS, saline infused sonography; SLL, single-layer continuous locked; TVUS, transvaginal ultrasound.

In another study, classical primary continuous suturing (CPCS) was compared with the novel technique uterine suturing (NTUS) comprising of a Z suture on both corners and eight sutures in the remaining middle part of the incision closure (39). A TVUS at 3 months post CS RMT showed a strong negative correlation with incision length after closure (*p* < 0.0001), number of sutures (*p* < 0.0001), and suturing time length (*r* = –0.237; *p* < 0.0001). The authors reported that the occurrence of uterine niche was similar in both groups.

In an RCT of 220 patients (40), double-layer interrupted vs. continuous sutures were compared. At follow-up, TVUS showed that interrupted sutures were superior in preventing severe CSD (2% vs. 22%, *p* < 0.0001). However, no significant difference in RMT was observed between the interrupted and continuous groups [median 8.1 (interquartile range, 6.2–9.9) mm vs. 7.9 (4.6–10.3) mm, respectively]. This was the first study to compare these techniques regarding niche formation. Other factors associated with severe CSD included the difference in myometrial thickness between the fundal and cervical sides at the center of the uterine wound and uterine retroversion.

### 3.5 Different suturing materials

#### 3.5.1 Catgut and synthetic

Historically, catgut suture, which is produced from bovine collagen has been widely used in gynecology and obstetrics, however, due to its pro-inflammatory properties and the increased availability and superiority of synthetic sutures, catgut popularity decreased (39).

The CORONIS study found no significant differences between chromic catgut and polyglactin-910 in maternal mortality, infectious morbidity, further operative procedures, or blood transfusion (>1 unit) up to 6 weeks postpartum (13, 14). Uterine scar assessment by gynecological ultrasound was not performed. The CORONIS study did not include a post-cesarean section gynecological ultrasound examination; thus, no data on the uterine scar parameters were available.

In another study, catgut sutures were associated with a higher niche rate than Vicryl sutures (18.2% vs. 9.3%, *p* = 0.03) at 6 months post-first CS, as assessed by TVUS (42). RMT was greater in the Vicryl group compared to the catgut suture group; however, the prevalence of postoperative gynecological sequelae such as postmenstrual spotting and pain was similar between the two groups.

#### 3.5.2 Monofilament and multifilament

Two RCTs assessed synthetic absorbable monofilament vs. multifilament sutures. In the study by Saccone et al. (43) including women undergoing their first or second CS, niche formation did not differ significantly between groups at 6 months (18.4% vs. 23.4%). There were no substantial differences found in the incidence of pelvic pain, dysmenorrhea, and dyspareunia. Başbuğ et al. (44) reported greater RMT with monofilament sutures at 6–9 months, without significant differences in gynecological sequelae.

#### 3.5.3 Suture absorption rates

In women undergoing their first CS, rapid-absorption Rapide Vicryl was associated with lower niche formation than conventional Vicryl at 5–6 months on TVUS (22.4% vs. 42.3%, *p* = 0.046), as well as less postmenstrual spotting (16.2% vs. 52.8%, *p* = 0.002) (45).

#### 3.5.4 Barbed sutures

In the SPIRAL RCT (46), women undergoing their first CS undergoing double-layer closure with continuous barbed sutures had a lower niche rate than those receiving conventional continuous polyglactin sutures at 4 months on TVUS (30.6% vs. 62.5%, *p* < 0.00), with greater RMT (8.46 vs. 7.07 mm). Barbed sutures also significantly improved niche length, depth, and width, with no cases of RMT <3 mm. No significant differences were observed in the operative times, maternal surgical complications, or postoperative complications. Interestingly, the niche rate was significantly affected by the surgeons’ years of experience (Table 5).

**Table 5:** Comparison of studies describing different suturing materials used for uterine closure.

Author/name of study	Year	Study type	Qualification criteria	Number of patients	Time of assessment	Method of assessment	Study objective	Results	Conclusions
Maki et al. (46)	2024	RCT SPIRAL trial	One CS	220	6 months	TVUS	Comparing uterine suture materials: barbed and conventional polyglactin sutures	Uterine niche rates: barbed suture group, 29.1% conventional suture group, 68.2% Barbed suturing significantly improved niche measurements length, depth and width and RMT; 8.46 vs. 7.07 mm	Double-layer barbed sutures during cesarean delivery may prevent CSD and postoperative complications RMT at 6 months post-surgery was significantly affected by both the surgeons’ years of experience and institutional factors
Yetkinel et al. (45)	2023	Retrospective	One CS	101	5–6 months post CS	TVUS	Comparing uterine suture materials: using a fast absorption suture material (Rapide Vicryl) and conventional sutures (Vicryl)	Uterine niche rates: Rapide Vicryl suture group, 22.4% Vicryl suture group, 42.3% (*p* = 0.046) Postmenstrual spotting was observed significantly lower in the Rapide Vicryl group (16.2%) vs. Vicryl group (52.8%) (*p* = 0.002)	The formation of niches and associated postmenstrual spotting rates were less frequent with suture materials that were absorbed faster
Saccone et al. (43)	2022	RCT	One or two CS	300	6 months post CS	TVUS	Comparing uterine suture materials: synthetic absorbable monofilament vs. multifilament sutures	Uterine niche rates: Monofilament, 18.4% Multifilament, 23.4% (*p* = 0.31) There was no between-group substantial difference found in the incidence of symptoms, including pelvic pain, painful periods, and dyspareunia	The use of synthetic absorbable monofilament sutures was not associated with a reduction of uterine niche at 6 months after delivery compared with the use of synthetic absorbable multifilament sutures
Hosseini et al. (42)	2021	Prospective clinical trial	One CS	250	6 months post CS	TVUS	Comparing uterine suture materials: vicryl sutures vs. catgut sutures	Uterine niche rates: Catgut, 18.2% Vicryl, 9.3% (*p* = 0.03) RMT was greater in the vicryl group compared to the catgut suture group	Vicryl sutures were associated with a lower risk of CSD formation in comparison with catgut sutures The prevalence of postoperative gynecological sequelae such as postmenstrual spotting and pain were similar between the two groups
Başbuğ et al. (44)	2019	RCT	One CS	107	6–9 months post CS	TVUS	Comparing uterine suture materials: synthetic absorbable monofilament vs. multifilament sutures	RMT was thicker in monofilament compared to multifilament suture group (5.5 ± 2.24 vs. 4.18 ± 1.76, *p* = 0.01)	Monofilament suture has a positive effect on scar healing and increases RMT thickness compared to multifilament sutures There was no statistically significant difference between the monofilament suture and multifilament suture groups in terms of gynecological sequelae.
CORONIS group (13, 14)	2013	RCT	One or two CS	15,935	Immediately after surgery, 6 weeks post CS	Telephone interview or home visit	Comparing uterine suture materials: chromic catgut vs. polyglactin-910	The primary outcome was the composite of death, maternal infectious morbidity, further operative procedures, or blood transfusion (>1 unit) up to the 6-week follow-up visit Both chromic catgut and polyglactin-910 for uterine repair are acceptable for uterine closure	There were no statistically significant differences within any of the intervention pairs for the primary outcome: chromic catgut vs. polyglactin-910 0.90 (CI 0.78–1.04)

CS, cesarean section; CSD, cesarean scar defect; RCT, randomized controlled trial; RMT, residual myometrial thickness; TVUS, transvaginal ultrasound.

## 4 Discussion

The heterogeneity of the included studies, including differences in eligibility criteria, follow-up duration, niche definition, imaging modality, surgical techniques, surgeon experience, and patient characteristics, limited the direct comparison of results and represents a major limitation of this review.

Most studies assessed patients 6 months after CS, with TVUS being the most frequently used diagnostic method, followed by SIS and GIS. Variability in imaging techniques likely contributed to the wide range of reported niche prevalence. Antila-Långsjö et al. (47) demonstrated that approximately 51% of niches detected by SIS were missed on TVUS, suggesting that SIS may represent the diagnostic gold standard (47).

The two largest RCTs, CAESAR and CORONIS, showed comparable maternal infectious morbidity following single- and double-layer uterine closure (13, 14, 28). Unfortunately, neither trial included ultrasound follow-up or evaluated niche formation or RMT, limiting their relevance for uterine niche outcomes.

The RCT 2Close trial and its follow-up study (16, 17), including 2,292 women, incorporated postoperative TVUS/GIS-SIS assessment and found no superiority of either closure technique, suggesting that surgeons may safely use their preferred method (17). The second-largest RCT, the NICEST trial (18), with uterine niche TVUS assessment at 6 and 12 months, demonstrated greater RMT after double-layer closure but no difference in niche incidence, while also showing that niche morphology continues to change during the first postoperative year.

The evidence from four studies (three RCTs and one retrospective study) suggests that endometrium-free uterine closure may reduce CSD incidence by preserving normal uterine tissue architecture (14, 15, 34–36), although one retrospective study found no significant advantage (37). In the 2Close trial, the choice of endometrial handling was left to the surgeon, introducing potential selection bias (16). The novel surgical techniques described in recent single-center studies (38–40) should be interpreted cautiously because of their higher risk of bias. Likewise, current evidence regarding suture absorption rates (41) and barbed sutures (46) remains insufficient to support firm recommendations, highlighting the need for large, multicenter randomized trials with standardized imaging protocols and long-term follow-up.

In addition to surgical factors, patient- and labor-related characteristics contribute to uterine niche development. Gestational diabetes, obesity, and multiple cesarean deliveries were found to increase the risk of uterine niche according to a large prospective cohort study by Antila-Långsjö et al. (47). Other important risk factors reported to influence uterine niche development include a low uterine incision, particularly through cervical tissue (48, 49); cesarean delivery during advanced labor (50); and uterine retroflexion (9). These factors, together with the surgical technique, influence uterine scar healing.

Asymptomatic uterine niches generally do not require treatment (51, 52). Symptomatic patients may benefit from medical or surgical management, including hysteroscopic, laparoscopic, or robotic repair, depending on symptoms, RMT, and reproductive plans (53–56). Laparoscopic repair has been associated with improved scar characteristics and reproductive outcomes (54, 55). Furthermore, preventive resection of the previous cesarean scar during repeat cesarean delivery has recently been reported to reduce niche incidence and severity without increasing operative morbidity, although further validation is required (57).

Given the increasing global rates of CS and CSD, there is an urgent need for high-quality, multicenter randomized controlled trials using standardized definitions, imaging protocols, and long-term follow-up to establish evidence-based recommendations for uterine closure techniques.

## 5 Conclusions

Cesarean scar defect is common after both primary and repeat cesarean delivery, and considerable efforts are focused on identifying surgical techniques and suture materials that optimize uterine healing and reduce uterine niche formation. Optimizing CS operative technique and suture materials may decrease the incidence of CSD and its associated complications, including cesarean scar pregnancy, abnormal placental implantation, and placenta accreta spectrum disorders.

Current evidence does not support the definitive superiority of one uterine closure technique over another for preventing uterine niche formation. Interpretation of the available evidence is limited by substantial heterogeneity in niche definitions, imaging methods, timing of assessment, surgical techniques, suture materials, operator’s experience, and patient characteristics.

Operative CS technique regarding single- and double-layer uterine closure remains inconclusive; however, a careful approximation of the hysterotomy wound is crucial, and most studies conclude that the final decision regarding the number of suture layers rests with the surgeon. Most studies suggest that unlocked suturing and endometrium-free closure are associated with improved postoperative outcomes. Furthermore, well-designed multicenter randomized controlled trials with standardized ultrasound follow-up are essential to determine the optimal approach to uterine closure. A careful qualification for cesarean section and continued education of both patients and healthcare professionals are essential to reduce CS-related morbidity.
