# Supplementary material for: Surgery-related factors predisposing to uterine niche after cesarean section: a systematic review
Source: Front Med (Lausanne). 2026 Oct 12;13:1838075. doi: 10.3389/fmed.2026.1838075 (PMC13696934; doi:10.3389/fmed.2026.1838075)
Supplement: Supplementary file 1 [file Datasheet1.pdf]

Appendix 1.

Table 1. Cochrane Risk of Bias 2.0 (RoB 2.0) framework was used to assess the risk of bias in randomized clinical trials and randomized prospective studies discussed in the review.

Author / Name of study	Cochrane Risk of Bias 2.0 (RoB 2.0) framework assessment: 1. Randomization process	Cochrane Risk of Bias 2.0 (RoB 2.0) framework assessment: 2. Deviations from intended interventions	Cochrane Risk of Bias 2.0 (RoB 2.0) framework assessment: 3. Missing outcome data	Cochrane Risk of Bias 2.0 (RoB 2.0) framework assessment: 4. Measurement of the outcome	Cochrane Risk of Bias 2.0 (RoB 2.0) framework assessment: 5. Selection of the reported result	Cochrane Risk of Bias 2.0 (RoB 2.0) framework assessment: Overall result
CORONIS group (12,13)	Low RoB	Low RoB	Low RoB	Low RoB	Low RoB	Low RoB
2Close study group (15,16)	Low RoB	Some concerns	Low RoB	Low RoB	Low RoB	Some concerns due to open-label character of RCT and possible co-intervention differences
Nicest study group (17)	Low RoB	Low RoB	Some concerns	Low RoB	Low RoB	Some concerns due to missing data due to loss to follow-up
Glavind (19)	Low RoB	Low RoB	Some concerns	Low RoB	Low RoB	Some concerns due to missing data and loss to follow-up
Bamberg (20)	Low RoB	Low RoB	Some concerns	Low RoB	Low RoB	High RoB due to missing data and major loss to follow-up
Yılmaz (22)	Some concerns	Low RoB	Some concerns	Low RoB	Some concerns	Some concerns due to allocation concealment, handling of missing data, and lack of a prespecified study protocol
Yıldız (23)	Some concerns	Some concerns	Low RoB	Low RoB	Some concerns	Some concerns due to unclear information about study protocol, trial registration and allocation concealment
Dimassi (24)	Some concerns	Low RoB	Low RoB	Low RoB	Low RoB	Some concerns due to limited information about of randomization and concealment methods
Halouani (26)	Some concerns	Low RoB	Low RoB	Low RoB	Low RoB	Some concerns due to limited information about of randomization process
CAESAR group (27)	Low RoB	Low RoB	Low RoB	Low RoB	Low RoB	Low RoB
Tekelioğlu (28)	Some concerns	Low RoB	Some concerns	Some concerns	Some concerns	High RoB due to limited information about of method of sequence generation, allocation concealment process, high attrition rate, blinding of sonographer was not clearly stated
Bayraktar (29)	Some concerns	Some concerns	Low RoB	Some concerns	Some concerns	Some concerns due to unclear information about study protocol, trial registration, allocation concealment and blinding of sonographers
Gumusburun (30)	Some concerns	Some concerns	Low RoB	Some concerns	Some concerns	Some concerns due to unclear information about study protocol, trial registration, allocation concealment and blinding of sonographers
Gezer (35)	Some concerns	Low RoB	Some concerns	Some concerns	Some concerns	High RoB due to limited information about of allocation concealment process, very high attrition rate, blinding of sonographer was not clearly stated
Mohr-Sasson (36)	Low RoB	Low RoB	Low RoB	Low RoB	Some concerns	Some concerns due to lack of explicit protocol registration
Kalem (37)	Some concerns	Low RoB	Low RoB	Some concerns	Some concerns	Some concerns due to unclear allocation concealment and randomization, unclear blinding of sonographers and no clearly accessible protocol
Tsuji (39)	Low RoB	Low RoB	Low RoB	Some concerns	Low RoB	Some concerns due to unclear information about blinding of sonographers
Saccone (42)	Low RoB	Low RoB	Low RoB	Some concerns	Some concerns	Some concerns due to unclear information about blinding of sonographers and no explicit publicly available study protocol
Basbug (43)	Low RoB	Low RoB	Low RoB	Low RoB	Some concerns	Some concerns due to unclear information about study protocol and trial registration
Maki (45)	Low RoB	Low RoB	Low RoB	Low RoB	Some concerns	Some concerns due to unclear information about study protocol and trial registration

Table 2. Newcastle–Ottawa Scale (NOS) assessment was used to assess the risk of bias and quality of retrospective and cohort studies discussed in the review.

Author / Name of study	Newcastle–Ottawa Scale (NOS) Assessment Score	Quality Interpretation	Commentary
Mehdiyev (25)	7/9	High quality	Limitations: comparability of cohorts and lack of robust multivariable adjustment reported
Meyer (34)	7/9	High quality	Limitations: non-randomized intervention allocation, residual confounding and possible selection bias
Tekiner (21)	8/9	High quality	Limitations: residual confounding risk
Antoine (33)	9/9	High quality	High methodological quality

Author / Name of study	Newcastle–Ottawa Scale (NOS) Assessment Score	Quality Interpretation	Commentary
Glavind (18)	9/9	High quality	High methodological quality
Erkayıran (38)	9/9	High quality	High methodological quality
Yetkinel (44)	8/9	High quality	Limitations: non-randomized intervention and residual confounding
Hosseini (41)	8/9	High quality	Limitations: residual confounding risk
